# P53 Dysfunction in Neurodegenerative Diseases - The Cause or Effect of Pathological Changes?

**DOI:** 10.14336/AD.2016.1120

**Published:** 2017-07-21

**Authors:** Aleksandra Szybińska, Wiesława Leśniak

**Affiliations:** ^1^Laboratory of Neurodegeneration, International Institute of Molecular and Cell Biology in Warsaw, 4 Ks. Trojdena St., 02-109 Warsaw, Poland; ^2^Department of Neurodegenerative Disorders, Laboratory of Neurogenetics, Mossakowski Medical Research Center Polish Academy of Sciences, 5 Pawinskiego St. 02-106 Warsaw, Poland; ^3^Department of Molecular and Cellular Neurobiology, Nencki Institute of Experimental Biology of the Polish Academy of Sciences, 3 Pasteur St., 02-093 Warsaw Poland

**Keywords:** p53, neurodegenerative diseases, Alzheimer disease, Parkinson disease, apoptosis, neuronal loss

## Abstract

Neurodegenerative diseases are a heterogeneous, mostly age-associated group of disorders characterized by progressive neuronal loss, the most prevalent being Alzheimer disease. It is anticipated that, with continuously increasing life expectancy, these diseases will pose a serious social and health problem in the near feature. Meanwhile, however, their etiology remains largely obscure even though all possible novel clues are being thoroughly examined. In this regard, a concept has been proposed that p53, as a transcription factor controlling many vital cellular pathways including apoptosis, may contribute to neuronal death common to all neurodegenerative disorders. In this work, we review the research devoted to the possible role of p53 in the pathogenesis of these diseases. We not only describe aberrant changes in p53 level/activity observed in CNS regions affected by particular diseases but, most importantly, put special attention to the complicated reciprocal regulatory ties existing between p53 and proteins commonly regarded as pathological hallmarks of these diseases, with the ultimate goal to identify the primary element of their pathogenesis.

P53 was discovered in 1979 and described as a protein involved in tumorigenesis [1, 2, 3]. Further studies revealed that it acts as a tumor suppressor and has been called “the guardian of the genome” [4]. Later, it was shown that it functions as a transcription factor [5, 6] that regulates numerous genes involved in such vital cellular pathways as those responsible for cell cycle control, DNA repair, cell metabolism, senescence, apoptosis or stress response [7]. The level and transcriptional activity of p53 massively increase in cells undergoing different types of stress including oxidative stress, DNA damage, telomere erosion or ribosomal stress [8]. This increase is a combined effect of transcriptional and post-transcriptional regulatory mechanisms, the latter involving a variety of p53 modifications: phosphorylation, acetylation, ubiquitination, neddylation, sumoylation, methylation and others, all of which affect p53 activity, localization and stability [9, 10]. For example, sequential phosphorylation at Ser15, Thr18, Ser20 stabilizes p53 by inhibiting its interaction with MDM2 (Murine double minute 2), which has ubiquitin ligase activity [11, 12, 13]. Moreover, phosphorylation at these residues facilitates acetylation in the C-terminus, which in turn regulates p53 oligomerization and nuclear transport, enhances DNA binding [14] and, in consequence, transcriptional activity. Phosphorylation of Ser315 may also lead to p53 activation by facilitating its binding to DNA [15, 16] although another study showed MDM2-dependent p53 ubiquitination and degradation following Ser315 phosphorylation by Aurora kinase A [17]. Phosphorylation at Thr377 and Ser378 clearly inhibits p53 acetylation and activity, promotes its ubiquitination by MDM2 and degradation [18].

Why do cells upregulate p53 when their homeostasis is threatened? All cells respond and adapt to external and internal stimuli including harmful ones. Depending on the gravity of the inflicted damage, environmental context and cell type the cell responds either by 1) temporal cell cycle arrest in order to repair the damage, 2) senescence, to prevent propagation of the damage to subsequent cell generations or 3) apoptosis, to eliminate damaged cells. As stated above, p53 controls genes involved in all these processes and is therefore decisive both for the choice of response and its intensity [19]. P53 can induce cell apoptosis by several mechanisms [20]. The most straightforward one is transcriptional activation of pro-apoptotic genes of the so called “intrinsic apoptotic pathway”, such as genes encoding proteins of the Bcl2 family or caspases, but transcription independent mechanisms of p53 action have also been described [21]. The ability of p53 to trigger apoptotic cell death has been harnessed to help kill tumor cells as most therapeutic strategies rely on p53 pro-apoptotic activity induced in response to cellular damage inflicted by anti-cancer drugs and procedures. Cell apoptosis, however, is not always desirable or beneficial and excessive apoptotic death can disturb the functioning of many tissues with limited regenerative potential. Indeed, dysregulated apoptosis has been recognized as a fundamental pathogenic mechanism in a number of human diseases characterized by cell loss and tissue degeneration, commonly referred to as degenerative diseases. Given the tight connection between p53 and apoptosis it is not surprising that the protein has been put under scrutiny. In particular, a concept has been proposed that p53 can be one of the pathogenic factors in neurodegenerative diseases, with a pivotal role in neuronal loss [22]. In this review, we consider multiple links connecting p53 with molecular symptoms of these diseases in order to decide over its causative or secondary involvement in this type of pathologies.

## Neurodegenerative diseases

Neurodegenerative diseases (ND) are a heterogeneous group of disorders characterized by progressive neuronal loss. The most prominent features which relate neurodegenerative diseases to one another are accumulation of protein aggregates and apoptotic death affecting neurons in different parts of the central nervous system (CNS), particularly in the brain. Differences usually concern the afflicted brain areas, pathological hallmarks and clinical symptoms.

Alzheimer disease (AD) is the most common age-associated neurological disorder. About 5% of all AD cases are familial (FAD), characterized by an early onset below the age of 65. FAD is associated with autosomal dominant mutations in 3 genes: encoding amyloid β precursor protein (APP) and the enzymatic components of γ-secretase complexes i.e., presenilin 1 (PS1) and presenilin 2 (PS2) [23, 24]. Most AD cases, over 90%, are sporadic (SAD), with no known associations with gene mutations, although apolipoprotein E4 gene allele is listed as a main risk factor [25]. Pathological hallmarks of both types of AD are neurofibrillary tangles formed by hyperphosphorylated tau, a protein belonging to microtubule associated proteins (MAPs), and extracellular plaques containing the amyloid beta 1-42 (Aβ_42_) fragment of APP, generated by aberrant activity of PS1/PS2. Despite many years of extensive studies AD pathogenesis is still elusive and, in consequence, there are no effective therapies or even precise early-stage diagnostic methods.

Parkinson disease (PD) is the second, in terms of the prevalence, neurodegenerative disorder after AD. It is characterized by progressive death of midbrain dopaminergic neurons of substantia nigra pars compacta [26] and the presence of intracellular inclusions called Lewy bodies consisting mainly of aggregated α-synuclein [27, 28]. Like in AD, most PD cases are sporadic. Familial cases are associated with mutations either in α-synuclein [29, 30, 31, 32, 33], LRRK2 (leucine-rich repeat kinase 2) [34, 35] or parkin [36]. Other genes associated with PD are PINK-1 (PTEN-induced kinase-1) [37] and DJ-1 [38]. Mutated α-synuclein is responsible for the autosomal dominant form of the disease, whereas mutations in parkin, PINK-1 and DJ-1 cause autosomal recessive PD.

Huntington disease (HD) is a genetic disorder caused by propagation of a glutamine (CAG) stretch within the N-terminal part of the huntingtin protein (Htt). Although mutated huntingtin (mHtt) is expressed ubiquitously, the disease symptoms mainly affect the medium spiny neurons of striatum and the large pyramidal neurons (layers III, V and VI) of the cerebral cortex [39]. A progressive dysfunction of the cortico-striatal connectivity coupled with degeneration and loss of striatal neurons lead to motor dysfunction, cognitive decline and psychiatric symptoms. At the cellular level, the disorder manifests itself in the form of nuclear and cytosolic inclusions containing aggregates of mHtt or its truncated N-terminal fragment. These pathological features are observed in postmortem HD brains [40, 41], neurons of HD transgenic mice [42] as well as in cultured cells engineered to express various forms of mHtt [43]. It is believed that mHtt aggregation is in fact a protective mechanism that helps to neutralize the apparently deleterious effect of the soluble protein [39].

Down syndrome (DS), or trisomy 21, is among the most frequent chromosomal abnormalities in human population. It is caused by the presence of an additional, third copy of chromosome 21 or its part. Features of DS are physical development delay, mild to moderate mental retardation, premature aging and development of AD-type neurodegeneration in relatively young individuals, the latter partially due to overexpression of AD associated genes located on chromosome 21 such as APP or superoxide dismutase 1 (SOD1) [44].

Amyotrophic lateral sclerosis (ALS) belongs to a group of progressive neurodegenerative diseases associated with death of upper and lower motor neurons, which leads to loss of voluntary muscles. Around 90-95% of cases are sporadic, with no known genetic basis. The remaining 5-10% are caused by mutations, and over 20% of inherited cases results from mutations in the gene encoding SOD1 [45].

Multiple sclerosis (MS) is either a progressive or relapsing - remitting autoimmune neurodegenerative disease in which immune cells cause myelin sheath disruption leading to focal lesions in brain and spinal cord. MS patients experience many autonomic, motor, visual and sometimes psychiatric problems. Although MS is not a hereditary disease there is a number of susceptibility variants within major histocompatibility complex (MHC), particularly in the human leukocyte antigen (HLA) region [46].

## P53 level and activity in neurodegenerative disorders

A substantial increase in p53 level and activity has been documented by numerous studies and seems to be a common feature of all neurodegenerative diseases [47]. In AD, increased p53 level was detected in various parts of patient brains [48, 49] when compared to brains of healthy individuals. Likewise, data from animal AD models showed an increase in p53 level in affected neurons [50]. As could be expected, higher p53 level was not without consequence for the cell. Multiple observations provided evidence that neurons in the brains of AD patients and of AD model animals were more sensitive to various stressors and underwent apoptotic death [51, 52, 53].

The same phenomenon i.e., increase in p53 level and activity was observed in PD patient brains as well as in PD animal and cellular models [54]. Here, again, altered expression and activity of p53 were associated with neuronal death. Immunostaining of affected patient brains revealed increased levels of inflammatory cytokines [54] as well as caspase 3 and Bax [55, 56], which together with DNA fragmentation and chromatin condensation indicated an apoptotic, p53 dependent mode of cell death [57, 58]. A substantially higher level of p53 was also detected in the affected brain areas of HD patients and disease animal models [40, 59] as well as in cells overexpressing mutated huntingtin [60]. As in AD and PD, increased p53 level correlated with DNA damage, activated cellular stress response and apoptosis [60]. A causal role of p53 in HD has been elegantly proved in a series of experiments on p53+/+, p53+/- and p53-/- mice transgenic for mHtt [39, 40]. Genetic deletion of p53 not only attenuated the cellular marks of mHtt expression, such as mitochondrial dysfunction, but also protected against neuronal degeneration and alleviated some of the neurobehavioral defects elicited by HD. Interestingly, even though p53 ablation did not prevent the formation of mHtt containing inclusions, p53-/- mice had lower mHtt level and increased aggregate load i.e. presented a milder disease phenotype [39].

Neurons of DS individuals also show increased susceptibility to apoptosis and there is experimental evidence that upregulation of p53 and other pro-apoptotic genes can be responsible for this. Altered expression of p53 and other apoptosis-related genes, e.g. Bax, GAP- 43, Fas, as well as an altered Bax/Bcl-2 ratio was demonstrated in brains and cultured neurons from DS patients and transgenic animal models [44, 47]. Quantitative ELISA (Enzyme-linked Immunosorbent Assay) revealed increased levels of p53 and of another pro-apoptotic protein, APO-1/Fas (CD95), in several regions of cerebral cortex and cerebellum of DS patients with AD-like neuropathology comparing to healthy controls [61].

In ALS, increased p53 immunoreactivity was observed in the motor cortex and spinal ventral horns of postmortem CNS tissues [62]. Immunoblotting and immunocytochemistry of CNS fragments showed not only elevated expression of p53, but also its differential localization in ALS patients. In control samples p53 was mainly expressed in non-neuronal cells, whereas in ALS the protein was found in motor neurons of the motor cortex and spinal cord, and its level was enhanced in astroglia [63]. Another study detected increased levels of p53 and other apoptotic markers (Rb, Bax, Fas and caspases) only in the spinal cord but not in the motor cortex of ALS individuals [64]. Higher p53 level was confirmed by qRT-PCR analysis of human material from ALS patients and age-matched controls. The same results were demonstrated by microarray and qRT-PCR analysis of dissected ventral horns from wobbler mice, an animal model of motor neuron death [65]. Activation of p53 and altered Bcl-x/Bax ratio, due to a decrease in anti-apoptotic Bcl-x and increase in pro-apoptotic Bax, was also observed in ventral horns of the lumbar spinal cord of mouse ALS models bearing mutated (G86R) SOD1 gene [66].

Increased p53 immunostaining and mRNA level were detected, already in the preclinical phase, in the retina of rats with myelin basic protein-induced experimental autoimmune encephalomyelitis (EAE), a commonly used animal model of MS that mirrors some aspects of the human disease including neuroinflammation, demyelination and neuronal death, particularly in retinal ganglion cells [67].

## Involvement of neurodegenerative disease-associated proteins in regulation of p53 level/activity

Experimental data gathered during the last decade provided an intriguing clue about possible involvement of proteins commonly associated with neurodegenerative disorders in regulation of p53 level and activity. In the case of AD pathology this has been broadly documented for presenilins i.e., enzymes that catalyze APP cleavage, APP and its proteolytic products, as well as tau. Conditional knock down of both PS1 and PS2 in mouse brain led to a decrease in p53 level and activity [68]. The same effect was observed in fibroblasts with PS1/PS2 deficiency and in cells treated with PS inhibitors or overexpressing catalytically inactive PS mutants [68]. These results corresponded with an earlier observation that PS2 overexpression triggered p53 dependent cell death [69] and suggested that APP and its proteolytic products may have an important role in the control of p53 level and activity. Indeed, lower level of p53 was observed in brains of APP-/- mice while studies on APP-/- fibroblasts confirmed that APP deficiency led to lower p53 mRNA and protein level and transcriptional activity [68]. A similar effect was observed in fibroblasts deficient in the amyloid-like precursor protein, APLP2 [68].

Interestingly, in contrast to the effect of PS2, overexpression of PS1 resulted in lower p53 level and activity, coupled to lower apoptosis rates, while PS1 deficiency yielded opposite effects [68]. Moreover, overexpression of other components of the γ-secretase complex, nicastrin [70, 81], Aph-1(anterior pharynx - defective 1) and Pen 2 (presenilin enhancer 2), also inhibited p53 in various cells and in AD animal models [71, 72]. This apparent discrepancy between the effect of PS1 (and other components of the γ-secretase complex) and PS2 with respect to p53 has been ascribed to the multiple complex interrelations existing between all these proteins, many of them involving reciprocal transcriptional regulation [73]. Altogether, these results indicate that proper physiological APP processing is intimately linked with p53 level and activity and, in consequence, with the plethora of cellular processes regulated by p53, to mention only apoptosis. An important question that follows is how this vital link is disturbed in AD pathology in which APP processing deviates from the physiological equilibrium. It is known for example that FAD-linked PS mutations selectively enhance production of the Aβ_42_ peptide, often at the expense of the less amyloidogenic Aβ_40_, and generate lower amounts of the APP intracellular domain (AICD) [74, 75]. In this respect, early studies conducted on APP-deficient rat neuroblastoma cells engineered to express wild type and FAD-mutant forms of APP showed that wt APP protected cells against apoptosis induced by UV irradiation, staurosporine or p53 activation [76]. The protection was due to strong inhibition of p53-DNA binding and therefore of p53-mediated gene transactivation. Interestingly, FAD-mutant APP did not show a protective effect [76]. Likewise, overexpression of various FAD-mutants of PS1 in HEK293 cells increased p53 transcriptional activity, in contrast to wt PS1 [68]. This result was corroborated by immunohistochemical analysis of p53 in brains of AD patients bearing these mutations [68]. Increased p53 and p21 levels were also characteristic for immortalized B-lymphocytes from FAD patients with a highly pathogenic P117R mutation in PS1 [77]. Thus, impairment of the physiological function of APP by mutations, or aberrant APP processing by mutated presenilins, result in higher p53 activity and could enhance neuronal susceptibility to stress and facilitate neuronal degeneration.

Like wt APP [76], wild type α-synuclein was shown to protect cells from pro-apoptotic stimuli in contrast to mutated A53T form of the protein associated with PD [69]. Wild-type α-synuclein lowered expression and transcriptional activity of p53 rescuing cells from apoptosis after staurosporine treatment [69]. Synphilin-1, a Lewy body component [78, 114], and a protein which interacts with α-synuclein, attenuated motor abnormalities and degeneration of brain neurons in mice bearing the A53T mutation in α-synuclein [79, 80]. Studies on cells overexpressing synphilin-1 have shown that it decreases p53 gene promoter activity, mRNA and nuclear p53 protein level [81]. Other genes involved in PD pathogenesis may also influence p53 activity. Namely, mutations in parkin, an E3-ubiquitin ligase [82, 83], which account for most autosomal recessive forms of juvenile PD, caused an increase in p53 mRNA level and consequent transcriptional activity [84]. Wild type parkin, on the other hand, protected cells (TSM1 neuronal cell line) from staurosporine induced apoptosis [84]. DJ-1 is another protein the mutations of which are linked to early-onset juvenile PD [38]. It is known as an oxidative stress sensor and chaperone protein [85, 86]. Studies involving either knock-out or overexpression of wt DJ-1 revealed that it repressed p53 transcriptional activity and in consequence diminished Bax expression in mammalian [87] as well as zebrafish PD models [88]. It has been shown recently that DJ-1 weakens the DNA binding affinity of p53 through direct interaction with its DNA-binding region [89].

Overexpression of mutated huntingtin in PC12 cells led to an increase in p53 level [40]. A similar causative relationship seems to link p53 with mutated SOD1. Mouse ALS models bearing G86R mutated SOD1 gene exhibited activation of p53 and a lower ratio of anti-apoptotic Bcl-x to pro-apoptotic Bax. PC12 cells overexpressing the same SOD1 mutated gene had elevated p53 expression and phosphorylation [66]. Human neuroblastoma SH-SY5Y cells expressing another SOD1 mutant, G93A, typical for familial ALS, also exhibited increased p53 activity and apoptotic rate [90]. Interestingly, resveratrol, a substance that increases expression of SIRT1 (Silent information regulator 1), an enzyme that deacetylates and in consequence reduces the activity of p53, delayed ALS onset and prolonged the lifetime of mice bearing the latter mutation. In multiple sclerosis activation of p53 and apoptotic cascade could be triggered by IL-1β, a proinflammatory cytokine [91]. Incubation of cortico-striatal slices of mice brain with IL-1β caused an increase in p21, a p53 target, as well as neuronal damage that could be abolished by p53 inhibitor pifithrin. Interestingly, Herold and coworkers [67], based on transcriptome analysis of retinal neurons from rat model of MS, concluded that the AD-associated protein, APP, could be involved in p53 activation at preclinical stage of the disease and that AICD could regulate p53 transcription in MS.

## Transcriptional and post-transcriptional control of p53 level and activity by proteins implicated in neurodegenerative diseases.

The tight correlation between an increased level of the protein hallmarks of ND and elevated p53 activity suggested that the former might directly or indirectly contribute to p53 activation. This indeed has been confirmed for example in the case of APP proteolytic products which were shown to regulate p53 gene transcription. Namely, AICD, the C-terminal intracellular fragment of APP, which can act as a transcriptional regulator [92], was shown to bind to and stimulate p53 gene promoter activity [68]. Furthermore, it appears that the extracellularly cleaved and deposited Aβ_42_ can be transported into the cell under oxidative and heat stress conditions and can directly activate the p53 gene promoter causing p53-dependent apoptosis [50]. In addition to p53, Aβ_42_ binds to promoters of other AD-associated genes such as BACE1 (beta-site amyloid precursor protein cleaving enzyme 1) and APP [93] and may intensify erroneous APP cleavage.

As mentioned above, parkin, one of the PD flagship proteins, appeared to attenuate p53 activity. This attenuation is most likely due to its transcription factor properties. It was shown that parkin could inhibit p53-dependent gene transcription by direct binding to the p53 gene promoter region [84]. Moreover, parkin bearing PD associated mutations binds p53 gene promoter less effectively and therefore fails to control p53 level/activity [84]. Thus, parkin mutations may contribute, at least in part, to the heightened cell death observed in a subset of PD cases. Interestingly, parkin transcription factor function is also implicated in AD, since it regulates transcription of genes encoding PS1 and PS2 [94].

It has been shown that Htt or its N-terminal portion can localize to the nucleus and participate in transcriptional regulation [95]. In particular, it was demonstrated that overexpression of mHtt, but not of wt Htt, led to transcriptional repression of p53-dependent genes such as p21 or multiple drug resistance-1 (MDR-1) [43].

One of the genes located on chromosome 21 and therefore overexpressed in DS is transcription factor ETS2. Studies performed on thymus cells and lymphocytes of ETS2 overexpressing mice as well as on ETS2 overexpressing HeLa cells demonstrated increase in p53 and its target genes levels and subsequent apoptosis. Crossing the ETS2 and p53-/- animals rescued this phenotype indicating, that ETS2-triggered apoptosis is p53 dependent [96]. Upregulation of p53 can be caused also by overexpression of another transcription factor encoded by a gene located in a Down syndrome region on chromosome 21, the homeobox protein Prep1 (PKNOX1). Fibroblasts from DS patients and murine cells overexpressing Prep1 proved to be more prone to genotoxic stress and subsequent apoptosis than control cells. This sensitivity was p53-dependent as p53 depletion largely attenuated the rate of etoposide-induced apoptosis [97]. Indeed, it was shown that p53 gene promoter was a direct target of Prep1.

P53 can also be activated in neurons of ALS patients with optineurin (OPTN) mutation. Wild type optineurin is a nuclear factor-kappa B (NF-κB) activity suppressor whereas its ALS-mutant is unable to inhibit NF-κB. Neuronal cells overexpressing mutated OPTN showed increased NF-κB activity and underwent cell death. As pro-apoptotic proteins such as p53 and Bax are downstream targets of NF-κB, its enhanced activity in OPTN-mutant cells likely triggers p53-dependent apoptosis [98].

A number of studies demonstrated that proteins associated with neurodegenerative diseases can contribute to disease progression not only by elevating the level of p53, but also by altering its posttranscriptional modifications. For example, DYRK1A (dual-specificity tyrosine-(Y) phosphorylation-regulated kinase 1A), encoded by a gene on chromosome 21, a Down syndrome critical region, was shown to phosphorylate p53 at Ser15 in vitro and in H19-7 rat embryonic hippocampal cells. Phosphorylation by DYRK1A resulted in p53 activation and elevation of its target genes expression, namely p21. Induction of p21 in H19-7 and in human embryonic stem cell-derived neural precursor cells affected G(1)/G(0)-S phase transition and decreased cell proliferation. The same was observed in embryonic DYRK1A-transgenic mouse brains. These results demonstrated that overexpression of DYRK1A may suppress neuronal proliferation via p21 induction by p53 [99]. However, DYRK1A can also have pro-survival activity. By phosphorylating its another target, SIRT1, it promotes p53 deacetylation and thus inhibition of pro-apoptotic genes [100]. Perturbations in p53 modifications were also observed in HD cellular models. Expression of mHtt in human neuroblastoma SH-SY5Y cells increased phosphorylation of p53 on Ser46 [59]. This, in turn, facilitated p53 interaction with the prolyl isomerase Pin1 and dissociation from iASPP, an apoptotic specific regulator of p53, allowing for expression of apoptotic target genes. In HEK 293 cells overexpression of mHtt affected upstream regulators of p53 such as CBP/p300 acetyltransferases and MDM2, leading to an increase in p53 Ser15 phosphorylation, decrease in Lys382 acetylation, alteration of p53 ubiquitination pattern and oligomerization activity [60].

Interestingly, mononuclear blood cells of AD patients, but not those suffering from PD or other type of dementia, were found to contain conformationally altered p53 [101]. This conformational mutant of p53 was also present in human neuroblastoma cells overexpressing wt APP and shown to arise due to nitration of tyrosine residues in response to oxidative stress. The mutant form was less active than wt p53 i.e., generated a milder response to acute toxic stress [102]. The same group earlier showed positive correlation between conformationally altered p53 and SOD activity in peripheral and immortalized cells of AD patients [103].

## The effect of p53 on expression, activity and other functional aspects of proteins implicated in neurodegenerative diseases

Many recent data provide evidence that p53 can regulate transcription of numerous genes whose products are directly implicated in the pathogenesis of neurodegenerative disorders. As for AD, p53 was shown to be a transcriptional repressor of PS1 [104, 105]. Taking into account that PS1 overexpression leads to a decrease of p53 level [68] this may represent a feedback regulatory mechanism controlling p53 activity. Obviously, this mechanism is disrupted once PS1 is mutated because, as mentioned before, mutated PS1 clearly stimulates p53 transcriptional activity [68].

p53 binds to promoters and activates transcription of parkin [106] and α-synuclein genes [94]. Again, as with PS1, there seems to be a reciprocal transcriptional control since both parkin and α-synuclein attenuate p53 transcription [69, 84]. Thus, a state of equilibrium is reached whereby increase in parkin or α-synuclein levels due to p53 activation leads to downregulation of p53 and to a subsequent decrease in parkin and α-synuclein transcription. Interestingly, p53 seems also to control, in an indirect parkin-dependent way, the level of DJ-1, another PD-linked gene. Duplan and co-workers [107] established that in brains of transgenic parkin deficient mice p53 decreases mRNA and protein levels of the X-box binding protein-1 (XBP-1), a transcription factor which stimulates DJ-1 expression. Since parkin regulates p53 level and activity, suppression of p53 leads to upregulation of XBP-1, which in turn binds and transactivates the DJ-1 gene promoter [107]. As mentioned above DJ-1 can bind to the DNA-binding domain of p53 and block its binding to gene promoters thus adding to the complexity of reciprocal interactions linking p53 with proteins involved in neurodegenerative disorders. Also, the huntingtin gene promoter contains putative p53 responsive elements and p53 binding to these sites has been shown to occur both in vitro and in vivo [39, 108]. P53 activation elicits an increase in Htt mRNA and protein levels. In particular, gamma irradiation increases Htt expression in the striatum and cortex of mouse brain in p53+/+ but not p53-/- animals. In fact, the level of Htt is lower in p53-/- mice [39]. Thus, the two proteins seem to act in a vicious regulatory cycle that probably exacerbates HD symptoms.

There are indications that p53 may be implicated and contribute to different neurodegenerative diseases not only by virtue of transcription factor activity but also through direct interactions with proteins implicated in these pathologies. Thus, in the case of AD pathogenesis, p53 involvement is not limited to a reciprocal regulatory cycle embracing presenilins and APP proteolytic products [72] but extends to the tau protein which, when hyperphosphorylated, forms neurotoxic neurofibrillary tangles [109]. It was observed that p53 could enhance tau phosphorylation in human cells [110] and that tau hyperphosphorylation was associated with neuroblastoma cell death [111]. The effect of p53 on tau hyperphosphorylation is indirect [110] and may be exerted through GSK3β (Glycogen synthase kinase 3β), which phosphorylates tau at sites implicated in AD [112]. p53 directly binds to and increases the activity of GSK3β while activated GSK3β phosphorylates and stimulates the transcriptional activity of p53 since inhibition of nuclear GSK3β attenuated p53-dependent transcription [113]. The link between p53 and GSK3β (i.e., between p53 and tau phosphorylation) may however be more complex since another study showed that GSK3β can regulate p53 levels through MDM2 phosphorylation, which is required for p53 degradation. In that case, inhibition of GSK3β should lead to an increase in p53 levels. Upon cellular stress, however, MDM2 levels diminish and in consequence association of GSK3β and p53 persists due to increased level of p53, and may possibly contribute to tau hyperphosphorylation [114].

**Table 1:** Involvement of p53 in neurodegenerative diseases.

Disease	p53-linked effects in neurons	Regulation of the level of ND-associated proteins by p53	Modulation of p53 level/activity by ND associated proteins
Alzheimer	DNA damage, activated stress response, apoptotic death of neurons [51-53]	PS1 down-regulation [104-105] GSK3β and phospho-tau upregulation [112]	PS2 - p53 upregulation [69] PS1 - p53 down- regulation, mut PS1- p53 upregulation [68] nicastrin, Aph1, Pen2- p53 down-regulation [70-72] APP, Aβ_42_, AICD - p53 upregulation [50, 68]
Parkinson	Apoptotic neuronal death [57-58] Caspase3, Bax levels increase [55, 56]	parkin [106] and α-synuclein [94] upregulation	α-synuclein, synphilin-1 - p53 down-regulation [69, 81] parkin - p53 down- regulation, mut. parkin -p53 upregulation [84] DJ-1 - p53 down- regulation [87]
Huntington	DNA damage, activated stress response, apoptotic neuronal death [60]	Htt upregulation [39, 108]	mut. Htt - p53 upregulation [40, 59]
Down syndrome	apoptotic neuronal death Bax, GAP-43, Fas levels increase [44, 47]		ETS2, Prep1 - p53 upregulation [96, 97]
ALS	Apoptotic neuronal death, Rb, Bax, Fas, caspase levels increase [64] Altered p53 localization [63, 64, 65]		mut SOD1, mutated optineurin - p53 upregulation [66, 90, 98]
MS	Apoptotic neuronal death [67, 91]		

In HD p53 was identified within mHtt aggregates and shown to interact with the N-terminal part of both wt and mHtt in vitro and in vivo [43]. The biological consequences of this interaction are not clear, however, p53-/- mice had increased mHtt aggregate load, which corresponded to milder disease phenotype [39]. Therefore, it might be speculated that p53 binding may somehow favor aggregate dissociation and mHtt release, which is equivalent to greater toxicity.

## Summary

**Figure 1.**
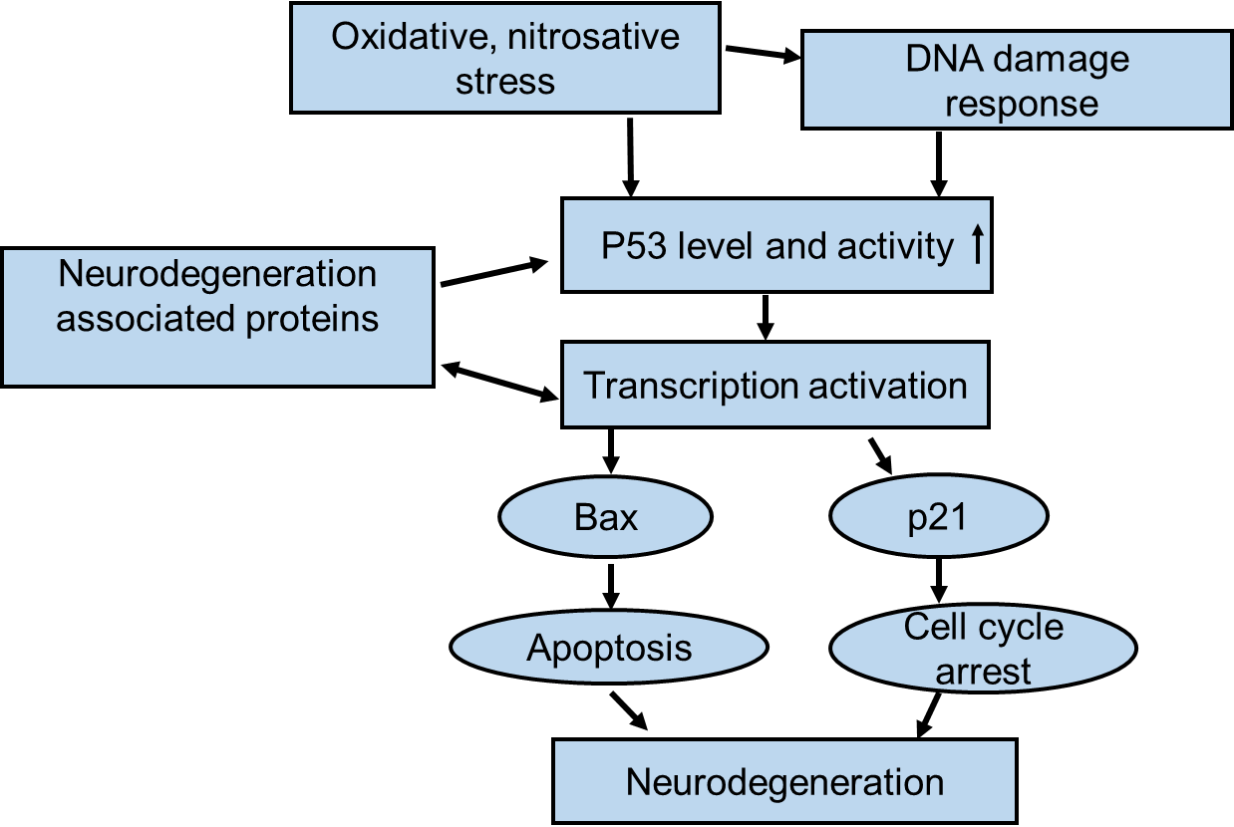
Simplified scheme of p53 involvement in neurodegenerative diseases p53 level and activity in neurons can increase not only as a result of oxidative stress and DNA damage but also due to aberrant regulation of its expression by mutated or erroneously cleaved proteins involved in neurodegeneration. Increased expression and activation of p53 entails enhanced expression of genes responsible for apoptosis or/and cell cycle arrest and, in consequence, may trigger neuronal death.

The reviewed data leave no doubt that p53 activation is one of discriminative molecular features of neurodegenerative diseases. Increased p53 level is infallibly detectable in brain areas attained by a particular disease, in the corresponding brain areas of animal models and in neuronal cells isolated from these brains. It is also evident that proteins commonly associated with ND pathology are involved in regulation of p53 level and activity as summarized in Table 1 and Figure 1. Mostly, it seems, in their wild type form, these proteins either directly or indirectly control and temper p53 activation, as was best illustrated in the case of parkin [84]. Keeping p53 at bay appears to be a difficult and precise task, which they apparently fail to fulfill when mutated or erroneously cleaved. Even worse, mutated APP, PS, Htt etc., seem to induce excessive p53 activation leading to apoptosis (Fig 1, Table 1). The complicated relation is reciprocal as p53 activates many genes encoding ND-associated proteins, including parkin. Importantly, p53 activation is not destined to exacerbate ND symptoms as it induces a number of genes that protect neurons exposed to various ND-associated toxicities from DNA damage or synaptic injury [115]. Apparently though, due to disturbed neuronal homeostasis, p53 activation does not serve its purpose but may even aggravate the symptoms.

Confronted with the causality dilemma which came first, p53 upregulation or molecular signatures of neurodegenerative diseases, one remains confused. An obvious track links p53 to oxidative stress - a common denominator of neurodegenerative diseases and an early event in their pathogenesis since its chemical signatures (e.g. free radicals, 0_3_) or biological consequences (e.g. DNA damage, mitochondrial dysfunction) are detectable before any macroscopic pathological changes can be observed. For example, in brains of transgenic mouse model (Tg2576) of AD, lipid peroxidation could be detected prior to amyloid plaque formation [116]. Also, cells expressing mHtt were shown to sustain DNA damage and exhibit mitochondrial dysfunctions before the appearance of mHtt aggregates and the same was noted in brains of HD patients and HD model mice [60]. DNA damage and DNA damage response were also observed in fetal and adult fibroblasts of DS patients [117]. Oxidative stress, and DNA damage that it provokes, are established, well defined signals for p53 activation. But again, the order of events remains obscure since the problem whether oxidative stress constitutes a primary cause of a neurodegenerative disease or is an aftereffect is still being debated [118, 119]. Functional defects caused by mutations within ND-associated proteins could conceivably disturb cellular homeostasis and provoke oxidative stress but apparently, most ND cases are sporadic, with no obvious associations with gene mutations. Thus, we still await elucidation of ND etiology and, with this, of the “rightful” place of p53 in it.
